# Weight Gain in Overweight and Obese People with HIV—The OBHIV Cohort

**DOI:** 10.3390/jcm13051211

**Published:** 2024-02-21

**Authors:** Lucia Taramasso, Silvia Dettori, Elena Ricci, Sonia Lerta, Sara Mora, Sabrina Blanchi, Mauro Giacomini, Antonio Vena, Matteo Bassetti, Antonio Di Biagio

**Affiliations:** 1 Infectious Disease Clinic, IRCCS Policlinico San Martino Hospital, 16132 Genoa, Italy; sabrina.blanchi@hsanmartino.it (S.B.); anton.vena@gmail.com (A.V.); matteo.bassetti@hsanmartino.it (M.B.); antonio.dibiagio@hsanmartino.it (A.D.B.); 2 Department of Health Sciences, Infectious Disease Clinic, University of Genoa, 16126 Genoa, Italy; silvia.dettori@hsanmartino.it (S.D.); lertasonia@gmail.com (S.L.); 3 Fondazione ASIA Onlus, Buccinasco, 20090 Milan, Italy; ed.ricci@libero.it; 4 Department of Informatics, Bioengineering, Robotics and System Engineering (DIBRIS), University of Genoa, 16126 Genoa, Italy; sara.mora@edu.unige.it (S.M.); mauro.giacomini@unige.it (M.G.)

**Keywords:** obese, overweight, OBHIV, weight gain, ART, comorbidities

## Abstract

Background: HIV and non-HIV-related factors have been related to weight gain (WG); however, their specific impact on people with HIV (PWH) who are overweight or obese remains unclear. Methods: This is a single-center observational study enrolling PWH with a BMI > 25 kg/m^2^. A generalized linear model was used to assess variables related to greater WG during 12 years of observation. Results: A total of 321 PWH were enrolled, 67% overweight and 33% obese, who gained an average of 0.2 ± 1.3 and 1.7 ± 1.5 kg/year, respectively (*p* < 0.0001). Years since HIV infection were the only variable significantly associated with WG (β −0.048, 95% CI −0.083; −0.013) during the study period, while type of ART did not influence the outcome. Narrowing the observation to the period of the SARS-CoV-2 pandemic, PWH with a longer duration of infection (β 0.075, 95% CI 0.033; 0.117) and a greater increase in triglycerides (β 0.005; 95% CI 0.000; 0.011) gained more weight, while higher BMI (β −0.256, 95% CI −0.352; −0.160), obesity (β −1.363, 95% CI −2.319; −0.408), diabetes mellitus (β −1.538, 95% CI −2.797; −0.278), and greater abdominal circumference (β −0.086, 95% CI −0.142; −0.030) resulted in protection. Conclusion: Among overweight and obese PWH, the amount of WG was higher in the first years after diagnosis of HIV and decreased thereafter, despite aging, regardless of the type of ART.

## 1. Introduction

In recent years, an increasing prevalence of overweight and obesity has been observed in people with HIV (PWH) [1]. Several factors have been related to weight gain in HIV, including environmental, genetic, and therapeutic [2]. However, it is unclear to date whether these factors have a different impact in underweight/normal-weight people, in whom increased body weight may be indicative of improved health and a return to well-being, and in people who are already overweight or obese, in which weight gain (WG) can be considered potentially detrimental on overall cardiometabolic health. In fact, in the general population, an increase in body mass index (BMI) has been shown to have negative implications in terms of survival in overweight or obese patients, whereas it is not related to unfavorable clinical outcomes in normal-weight or underweight subjects [3]. In contrast, a survival benefit has been observed in underweight people who increase their BMI, which has been confirmed in the specific setting of HIV infection [4,5,6]. It follows that the heterogeneity of PWH enrolled in studies focused on WG available to date may be an important limitation in the interpretation of the results. Indeed, overweight and obese people are on average less affected by body weight fluctuations during antiretroviral therapy (ART) than those with lower BMI levels [4,7], and a higher BMI seems to be even protective toward ART-associated WG [8]. Consequently, peculiar factors related to weight gain in this population are likely to be poorly identifiable in studies involving also underweight and normal-weight people, who on average have significantly greater weight changes compared to them. This implies that clinical studies on WG may be skewed toward identifying factors related to return to wellness and may be less informative about what factors are linked to undesirable weight gain in people already diagnosed as overweight or obese, who are precisely those to whom the clinical question about what factors, including the type of ART, may be harmful is most relevant. The hypothesis underlying the present study is that the epidemiological, therapeutic, and immuno-virological factors associated with weight gain in overweight or obese people may be peculiar and not generalizable to all PWH, and therefore, a focused study is required in a selected population.

This study aimed to create a cohort of Overweight and obese people with HIV (OBHIV cohort) and to describe their WG across years, as well as factors related to greater weight gain.

## 2. Materials and Methods

This study is a single-center retrospective cohort study. All PWH who consecutively visited the outpatient service of IRCCS Policlinico San Martino Hospital in Genoa between January 2021 and January 2022, with a BMI > 25 kg/m^2^ and who provided valid consent for participation, were enrolled in the OBHIV (overweight and obese people living with HIV) cohort. IRCCS Policlinico San Martino is a tertiary care university hospital in Genoa, northern Italy, where about 1300 PWH are supported in the outpatient service. Data were retrospectively collected from 12 years of follow-up prior to enrollment, or from the time of initiation of the first ART for those with a follow-up of less than 12 years.

Each participant was given a numerical anonymous code to ensure confidentiality and privacy in the processing of sensitive data.

The following variables were assessed: demographic factors (gender, age, and ethnicity); risk factor for acquisition of HIV infection; anthropometric parameters (weight, height, abdominal circumference, and BMI); metabolic parameters (total cholesterol, HDL cholesterol, LDL cholesterol, and triglycerides); immune-virological staging (stage and year of HIV infection, zenith of HIV-RNA, nadir of CD4+ T lymphocytes, and cumulative HIV viremia of the past 10 years expressed as viremia copy years, VCY); risk factors for the development of cardio-metabolic disease (cigarette smoking, blood pressure, and previous cardiovascular or cerebrovascular events); comorbidities (diabetes, metabolic syndrome, and psychiatric disorders); time of exposure to different antiretroviral drugs and classes (tenofovir disoproxil fumarate, TDF, tenofovir alafenamide, TAF, lamivudine/emtricitabine, XTC, abacavir, ABC, non-nucleoside reverse transcriptase inhibitors, NNRTIs, protease inhibitors, PIs, and integrase inhibitors, INSTIs); dietary habits, assessed by the questionnaire from the National Research Institute for Food and Nutrition (Istituto Nazionale di Ricerca per gli Alimenti e Nutrizione, INRAN); physical activity, assessed by the international physical activity questionnaire (IPAQ); sleep quality, assessed by the Pittsburgh Sleep Quality Index (PSQI) questionnaire.

Overweight was defined as BMI > 25 and ≤30 kg/m^2^, while obesity was defined as BMI > 30 kg/m^2^ at study entry. The past viral burden was measured for each participant as VCY, a measure of the cumulative plasma burden of HIV-RNA that corresponds to the number of copies of HIV RNA per mL to which a person is exposed over time. For example, 10,000 copy-years of HIV RNA may indicate exposure for one year to a viremia of 10,000 copies/mL or 10 years of a viremia of 1000 copies/mL. The trapezoidal rule is used to calculate VCY, by which the integral representing the area under the drawn curve of viral loads collected longitudinally over time for each patient is approximated [9]. The area under the curve of the segment between each pair of consecutive viral loads was calculated by multiplying the average of the two loads by the time interval between the measurements. The viral burden for each person was finally obtained by the sum of the area under the curve of all segments considered. All viral loads (HIV RNA) available for each patient from the date of the first diagnosis of HIV infection to the date of enrollment in the study, up to a maximum observation time of 10 years, were used for this calculation.

### 2.1. Study Objectives

The primary objective of this study was to assess the annual weight gain in a cohort of overweight or obese PWH and to evaluate the association of greater weight gain with demographic and viro-immunologic factors and therapeutic exposure to different antiretroviral drugs and drug classes.

Secondary objectives were to compare annual weight gain, comorbidity prevalence, and lifestyle habits (smoking, dietary habits, sleep quality, and grade of physical activity) among overweight and obese PWH and to compare weight trajectories before and after the SARS-CoV-2 pandemic to verify possible differences linked to the lifestyle modification and psychological implications resulting from the pandemic [10,11,12].

### 2.2. Statistical Methods

Data were described using mean and standard deviation (SD) for normally distributed continuous variables, median and interquartile range (IQR) for non-normally distributed continuous variables, and frequency (%) for categorical and ordinal variables. Distribution normality was assessed using the graphical quantile–quantile method. Baseline differences between means were tested using the analysis of variance and between medians using the non-parametric Mann–Whitney test. Proportion comparisons were performed using the chi-square test. The annual weight change in overweight and obese PWH was described as the mean increase in kg/year (±SD). The correlation between annual WG and other variables was assessed using a generalized linear model. A generalized linear model was also used to assess the correlation between mean weight change before and during the SARS-CoV-2 pandemic and the study variables. In multivariate models, only variables with *p*-value < 0.05, as determined by univariate analysis, were included. All statistical analyses were performed using SAS for Windows 9.4 (SAS Institute, Cary, NC, USA).

In the two subgroups of overweight and obese PWH, lifestyle, dietary habits, sleep quality, and daily physical activity were described graphically using bars or pie charts.

The study protocol was approved by the Ligurian Ethics Committee (registry number 254/2021). Written consent for study participation was obtained from all participants, and the study was conducted in accordance with the ethical standards of the 1964 Declaration of Helsinki and Italian national laws.

## 3. Results

### 3.1. Study Participants

A total of 321 PWH were enrolled in the OBHIV cohort, of which 214 (66.7%) were diagnosed as overweight and 107 (33.3%) as obese. The mean age was 53.7 (±10.3) years, and 91/321 (28.4%) were female. Overweight and obese PWH had similar characteristics, lifestyle habits, and comorbidities at baseline, except for a significantly higher proportion of hypertension and metabolic syndrome among the obese (61.7% and 37.4%, respectively) than those who were overweight (45.3% and 20.6%, respectively). In addition, obese PWH had higher HIV RNA zenith than overweight PWH, with a median value of 4.87 (IQR 3.98–5.48) log_10_ vs. 4.69 (IQR 3.09–5.26) log_10_, respectively. The annual weight gain observed over the 12 years of the study was 0.7 ± 1.5 kg/year, with significantly higher values in obese (1.7 ± 1.5 kg/year) than in overweight PWH (0.2 ± 1.3 kg/year), *p* < 0.0001. PWH who were obese at study entry (2021) also had higher BMI values at ART initiation/at the first available follow-up compared to overweight PWH (BMI 29.6 ± 4.43 vs. 25.2 ± 2.78 kg/m^2^ respectively, *p* < 0.001). The complete characteristics of the study population are presented in Table 1, Tables S1 and S2.

### 3.2. Factors Associated with Greater Weight Gain in Overweight and Obese People

Univariate analysis revealed a positive association between weight gain and VCY, and between weight gain and HIV RNA zenith, while factors that showed a negative correlation were increasing age, years of HIV infection, and years of exposure to drugs in the NNRTI and PI classes, as well as to TDF and XTC.

After adjustment in the multivariable analysis, only the total number of years of HIV infection maintained a statistically significant association with body weight, and as the number of years of infection increased, the magnitude of annual weight gain decreased (β −0.048, 95% CI −0.083; −0.013; *p* value = 0.007).

None of the individual antiretrovirals or ART classes considered showed an association with WG trends after multivariable adjustment. Table 2 presents the complete results of the analysis.

### 3.3. Weight Trend during the SARS-CoV-2 Pandemic

Two hundred and eighty-eight PWH had available follow-ups both before (January 2010–February 2020) and during (March 2020–January 2022) the SARS-CoV-2 pandemic period. Weight gain was 1.37 kg/year in the pre-pandemic period and 1.15 kg/year during the pandemic (*p* = 0.30). Obese PWH, but not overweight PWH, had lower weight gain during the SARS-CoV-2 pandemic (Table 3).

People who experienced greater weight gain during the pandemic were those with a longer duration of HIV infection (β 0.075, 95% CI 0.033; 0.117) and a greater increase in triglyceride values (β 0.005; 95% CI 0.000; 0.011) during the same period. In contrast, greater pre-pandemic BMI (β −0.256, 95% CI −0.352; −0.160), diagnosis of metabolic comorbidities (obesity, β −1.363, 95% CI −2.319; −0.408, and diabetes mellitus, β −1.538, 95% CI −2.797; −0.278), and greater abdominal circumference (β −0.086, 95% CI −0.142; −0.030) were associated with lower weight gain during the pandemic compared with the pre-pandemic period. Ongoing ART during the pandemic had no impact on the differences between the weight trends in the two periods considered (Table 4).

### 3.4. Lifestyle and Sleep Quality in Obese and Overweight PWH

The lifestyle habits of the participants in the OBHIV cohort were studied by administering questionnaires on eating habits (INRAN questionnaire), physical activity (IPAQ), and sleep quality (PSQI questionnaire).

A total of 48 PWH (21 diagnosed with obesity and 27 with overweight) agreed to complete the questionnaires. PWH diagnosed with obesity generally reported a lower frequency of consumption of most of the food classes investigated when compared to overweight people. The frequency of intake of the different food categories investigated using the INRAN questionnaire is shown in Supplementary Figure S1.

Among obese and overweight PWH, 9/21 (42.8%) and 11/27 (40.7%) reported an active/very active lifestyle (Met ≥ 2520); 3/21 (14.3%) and 5/27 (18.5%) reported a sufficiently active lifestyle (Met of 700–2519); and 6/21 (28.6%) and 8/27 (29.6%) reported an inactive lifestyle (Met < 700), respectively (Supplementary Figure S2).

Sleep quality was disturbed in 32/48 (66.6%) participants, including 14/21 (66.6%) obese and 18/27 (66.6%) overweight participants (Supplementary Figure S3).

## 4. Discussion

This study specifically approached the issue of weight gain in PWH diagnosed as overweight and obese, creating the first pilot cohort of people (OBHIV cohort) at a higher risk of developing metabolic and cardiovascular consequences than the normal-weight population [3]. This differentiation is important because several studies have investigated factors associated with WG [2,13,14,15], but it is still unclear whether specific and different factors should be considered when initiating follow-up or treatment in already overweight or obese individuals. In particular, it is still under investigation whether overweight and obese PWH should be preferably treated with ART regimens that do not include drugs associated with greater weight gain in the literature [8,15,16,17]. In OBHIV, the cumulative exposure to different antiretrovirals and ART classes has been specifically studied, but no significant correlations with WG were found after multivariable correction, even if a trend toward a protective effect of TDF, PI, and NNRTI classes was observed in the univariate analysis, probably reflecting the use of these drugs in earlier study years and thus identifying PWH with a longer history of HIV infection. The duration of HIV infection was the only factor that was significantly associated with WG in OBHIV. The maximum annual WG was observed in the first few years after the diagnosis of HIV, while a lower weight gain occurred thereafter, over a total of 12 years of follow-up. This trend suggests a significant impact of the initiation of therapy in terms of a return to well-being or, at least, a return to a lifestyle that is no longer conditioned by the symptoms of uncontrolled infection. In fact, a lifestyle free of disease conditioning might also be associated with unhealthy habits and thus lead to emerging overweight or obesity. Less likely is the role of individual antiretrovirals, which, if present and justified by a clear pathogenetic mechanism, should continue to have an impact throughout the period of exposure to the same drug, barring mechanisms of desensitization or adaptation. In accordance with this hypothesis, other studies have also found a higher WG in the first months after the start of a given ART regimen and a subsequent plateau thereafter [18,19,20,21]. PWH who were obese at study entry in 2021 had a greater weight gain over the 12 years of the study, but they also had a greater BMI at the first observation retrospectively collected. This observation suggests an already different situation at baseline and possibly different lifestyle habits in obese individuals who were already heavier than overweight PWH at the beginning of the observation period and experienced further weight increase thereafter. In fact, the magnitude of weight increase was not correlated with any of the clinical, therapeutic, and immuno-virological factors studied in the multivariate analysis, suggesting a major role of factors unmeasured in this analysis, such as diet and physical activity [22].

In the OBHIV cohort, lifestyle habits were also investigated using questionnaires, which did not reveal major differences between obese and overweight PWH. However, only a subgroup of study participants answered the questionnaires and only a self-evaluation without external control was performed, possibly limiting the reliability of the obtained answers. Less than half of the respondents reported an active lifestyle, suggesting the possibility of improvement with lifestyle interventions in this area [23]. Regarding eating habits, the questionnaire investigated the weekly frequency (but not food portion size) of different food categories. Obese PWH reported eating almost all food categories less often than overweight PWH, including healthy foods such as fresh fish, fruits, and vegetables. This type of response might reflect a diet further away from the diet considered optimal for cardiovascular risk control [24], but inaccuracy in data compilation or poor awareness of dietary habits is also possible. In addition, in OBHIV, more than half of the PSQI respondents reported disturbed sleep quality, which in turn has been correlated in the literature with states of anxiety and altered eating behavior. In fact, sleep restriction has been correlated with increased carbohydrate [25,26] and fat intake [27,28], and partial sleep restriction has been associated with reduced physical activity [29], increased caloric consumption, and significant weight gain [30]. This observation might, again, suggest the need for lifestyle interventions, but also for the assessment of the actual and perceived sleep quality elements that have not yet been implemented to date in the approach to the WG phenomenon in PWH.

Finally, we investigated whether differences in weight trends were observed after the SARS-CoV-2 pandemic began. In the general population, the pandemic period and the implementation of lockdown measures have been associated with greater weight gain than expected, to the point of a new phenomenon called “Covibesity” by experts [10,11,12]. However, the same was not observed in the OBHIV cohort. One possible explanation may be that the pandemic period considered in our study was not limited to the months of lockdown, but to a broader period of almost two years, in which any rapid weight change may not have been captured at the clinical visit and diluted in the calculation of the average trend over the total two years of the pandemic. Another important consideration is that the OBHIV cohort included only people who already had a diagnosis of overweight or obesity, who therefore might already have had suboptimal lifestyle habits even before lockdown-related conditioning. In fact, obese PWH and those with metabolic comorbidities were less likely to have increased WG during the pandemic period. In addition, it is possible that motivational interventions, medical counseling, and awareness of the diagnosis may have collaborated to put in place protective mechanisms against WG.

The limitations of this study include its retrospective and observational design, lack of objective measures of lifestyle habits, and the single-center evaluation.

## 5. Conclusions

In the OBHIV cohort, we found a progressive average weight gain over the 12 years of observation, greater in PWH with obesity, and not related to ART exposure, nor to clinical or immunovirological data investigated in the cohort, but only inversely related to years of HIV infection. This might suggest a major role of baseline lifestyle habits, even before HIV infection, and possible resumption of the same after the initiation of effective ART. Expanding the study to other centers will be critical to confirm these findings in a numerically larger and more heterogeneous sample. If confirmed and validated, the results of this study would have important clinical implications. On the one hand, therapies with modern antiretrovirals, which have been associated with weight gain in other clinical settings, would not be precluded for obese or overweight people, with the knowledge that they would not affect long-term weight outcomes. On the other hand, awareness of the role of risk factors can lead to targeted interventions. Last but not least, when an obese or overweight person starts treatment, the physician may be able to provide specific information about the risk of further weight gain in that specific context, instead of having to draw on more general data, in a concept of progressive personalization of clinical approach and counseling.

## Figures and Tables

**Table 1:** General characteristics of the OBHIV (overweight and obese people living with HIV) cohort and comparison of people diagnosed with overweight (n = 214) or obesity (n = 107).

Participants n (%)	Total n = 321 (100)	Overweight n = 214 (66.7)	Obese n = 107 (33.3)	*p* Value
Clinical characteristics				
Females, n (%)	91 (28.4)	58 (27.1)	33 (30.8)	0.48
Mean age (±SD)	53.7 (±10.3)	53.7 (±10.4)	53.8 (±10.1)	0.91
Caucasian, n (%)	273 (85.0)	180 (84.1)	93 (86.9)	0.51
HIV-RNA < 50 copies/mL, n (%)	308 (96.0)	207 (96.7)	101 (94.4)	0.38
Median CD4+ lymphocytes cells/mm^3^ (IQR)	690 (479–913)	714 (483–932)	662 (449–867)	0.29
First BMI measured in 2010 or at ART initiation (kg/m^2^), mean (±SD)	26.1 (±4.03)	25.2 (±2.78)	29.6 (±4.43)	<0.001
BMI 2021 (kg/m^2^), mean (±SD)	29.5 (±4.3)	27.1 (±1.4)	34.2 (±4.3)	-
Weight 2021 (kg), mean (±SD)	86.8 (±14.6)	80.4 (±9.4)	99.6 (±14.8)	-
Mean weight change 2010–2021 (kg/years), mean (±SD)	0.7 (±1.5)	0.2 (±1.3)	1.7 (±1.5)	<0.0001
Comorbidities, n (%)				
Diabetes	38 (11.8)	21 (9.8)	17 (15.9)	0.11
Hypertension	163 (50.8)	97 (45.3)	66 (61.7)	0.006
Metabolic syndrome	84 (26.2)	44 (20.6)	40 (37.4)	0.001
Fatty liver disease	103 (32.1)	63 (29.4)	40 (37.4)	0.15
Lipodystrophy	10 (3.1)	7 (3.3)	3 (2.8)	0.82
Stroke	7 (2.2)	3 (1.4)	4 (3.7)	0.18
Myocardial infarction	11 (3.4)	8 (3.7)	3 (2.8)	0.66
Psychiatric disorder	60 (18.7)	38 (17.8)	22 (20.6)	0.54

BMI: body mass index; IQR: interquartile range; n: number of observations; SD: standard deviation.

**Table 2:** Univariate and multivariable analysis of factors associated with greater annual weight gain in the study population.

	Univariate Analysis				Multivariable Analysis			
	β	95% CI		*p* Value	β	95% CI		*p* Value
		Lower	Higher			Lower	Higher	
Female Sex	−0.342	−0.920	0.236	0.25				
Age (by 1 year)	−0.046	−0.049	−0.043	0.0003	−0.016	−0.043	0.011	0.26
Copy-Years Viremia (by 1 log_10_ copies/mL)	0.259	0.112	0.406	0.0006	0.064	−0.132	0.260	0.52
Zenith HIV-RNA (by 1 log_10_ copies/mL)	0.216	0.053	0.379	0.01	0.100	−0.106	0.310	0.34
Nadir CD4+ (ref. < 200 cells/mm^3^)	0.48	−0.030	0.990	0.068				
CD4+ yearly increase (by 25 cells/mm^3^)	0.040	−0.015	0.095	0.16				
Years of HIV infection (by 1 year)	−0.075	−0.099	−0.051	<0.0001	−0.048	−0.083	−0.013	0.007
Years of NNRTI (by 1 year)	−0.087	−0.140	−0.034	0.0016	−0.018	−0.085	0.049	0.59
Years of PI exposure (by 1 year)	−0.071	−0.116	−0.026	0.0027	−0.008	−0.067	0.051	0.79
Years of INSTI exposure (by 1 year)	−0.019	−0.097	0.059	0.64				
Years of TDF exposure (by 1 year)	−0.088	−0.133	−0.043	0.0002	−0.027	−0.090	0.036	0.39
Years of TAF exposure (by 1 year)	0.074	−0.059	0.207	0.28				
Years of ABC exposure (by 1 year)	0.082	−0.004	0.168	0.063				
Years of XTC exposure (by 1 year)	−0.098	−0.139	−0.057	<0.0001	−0.009	−0.076	0.058	0.79
Total Cholesterol (by 10 mg/dL)	−0.046	−0.113	0.021	0.18				
HDL Cholesterol (by 10 mg/dL)	−0.126	−0.306	0.054	0.17				
LDL Cholesterol (by 10 mg/dL)	−0.014	−0.096	0.068	0.74				
Triglycerides (by 10 mg/dL)	−0.021	−0.052	0.010	0.19				
Fasting glucose in non-diabetic (by 10 mg/dL)	−0.001	−0.173	0.171	0.98				

95% CI: 95% confidence interval; ABC: abacavir; INSTI: integrase inhibitors; NNRTI: non-nucleoside reverse transcriptase inhibitors; PI: protease inhibitors; TAF: tenofovir alafenamide; TDF: tenofovir disoproxil fumarate; XTC: lamivudine/emtricitabine.

**Table 3:** Comparison of weight gain before (January 2010–February 2020) and during SARS-CoV-2 pandemic period (March 2020–January 2022) in the OBHIV (overweight and obese people living with HIV) cohort.

Study Population	Pre-Pandemic Period	SARS-CoV-2 Pandemic Period	Delta WG Pandemic–Pre-Pandemic	95% CI	*p* Value
All (n = 288)	1.37 ± 2.67	1.15 ± 2.66	−0.22 ± 3.68	−0.65; 0.20	0.30
Overweight in 2019 (n = 212)	1.11 ± 1.89	1.27 ± 2.18	0.16 ± 2.98	−0.24; 0.56	0.43
Obese in 2019 (n = 76)	2.12 ± 4.05	0.83 ± 3.67	−1.29 ± 5.03	−2.44; −0.14	0.028

**Table 4:** Univariate and multivariable analysis of factors associated with greater weight gain in a cohort of overweight and obese people living with HIV (OBHIV cohort) during the SARS-CoV-2 pandemic, considering the difference in gain between the pre-pandemic and pandemic periods.

	Univariate				Multivariable			
		95% CI				95% CI		
	β	Lower	Higher	*p*	β	Lower	Higher	*p*
Sex	0.128	−0.809	1.065	0.79				
Ethnic group	−1.102	−2.290	0.086	0.07				
Age (by 1 year)	0.036	−0.007	0.079	0.11				
2010 BMI	−0.121	−0.284	0.042	0.14				
2019 BMI	−0.287	−0.381	−0.193	<0.0001	−0.256	−0.352	−0.160	<0.001
Obese in 2019 (BMI ≥ 30 kg/m²)	−1.451	−2.402	−0.500	0.003	−1.363	−2.319	−0.408	0.0055
Current smoker	0.788	−0.190	1.766	0.12				
Former smoker	−0.224	−1.324	0.876	0.69				
Current alcohol abuse	0.446	−0.446	1.338	0.33				
Current drug abuse	1.638	−1.131	4.407	0.25				
Lipodystrophy	−0.038	−2.484	2.408	0.97				
Diabetes	−1.882	−3.136	−0.628	0.0035	−1.538	−2.797	−0.278	0.017
Hypertension	−0.335	−1.188	0.518	0.44				
Metabolic syndrome	−0.607	−1.562	0.348	0.21				
New metabolic syndrome in period 2	−0.749	−2.011	0.513	0.25				
Steatosis	0.115	−0.847	1.077	0.81				
New steatosis in period 2	0.21	−1.393	1.813	0.8				
Psychiatric illness on treatment	−0.35	−1.459	0.759	0.53				
Abdominal circumference	−0.081	−0.134	−0.028	0.0036	−0.086	−0.142	−0.030	0.003
Current ART								
INI	−0.383	−1.238	0.472	0.38				
PI	0.422	−1.119	1.963	0.59				
NNRTI	0.33	−0.525	1.185	0.45				
TAF	0.816	−0.166	1.798	0.1				
TDF	0.725	−6.513	7.963	0.84				
Dual therapy								
Nadir CD4+	−0.226	−1.079	0.627	0.6				
Years of HIV infection (by 1 year)	0.084	0.043	0.125	0.0001	0.075	0.033	0.117	0.0005
Glucose over time	0.003	−0.021	0.027	0.78				
Total cholesterol over time	0.007	−0.005	0.019	0.29				
Cholesterol LDL over time	0.005	−0.015	0.025	0.64				
Triglycerides over time	0.007	0.001	0.013	0.023	0.005	0.000	0.011	0.066

Factors with *p* < 0.05 in univariate analysis were included in the multivariable model. BMI 2019, obese 2019, and abdominal circumference were included in turn in the multivariable model. 95% CI: 95% confidence interval; BMI, body mass index; CI, confidential interval; HIV, human immunodeficiency virus; INI, integrase inhibitors; LDL, low-density lipoprotein; NNRTI, non-nucleoside reverse transcriptase inhibitors; PI, protease inhibitors; TAF, tenofovir alafenamide; TDF, tenofovir disoproxil fumarate.

## Data Availability

All data are available from the corresponding author upon reasonable request.

## Supplementary Materials

The following supporting information can be downloaded at: https://www.mdpi.com/article/10.3390/jcm13051211/s1, Table S1: Immuno-virological variables and antiretroviral treatment history in the OBHIV (overweight and obese people living with HIV) cohort and comparison of characteristics of people diagnosed with overweight (n = 214) or obesity (n = 107). Table S2: Blood parameters of glucose and lipid metabolism and lifestyle habits of the OBHIV (overweight and obese people living with HIV) cohort and comparison of people diagnosed with overweight (n = 214) or obesity (n = 107). Figure S1: Number of servings/week consumed for different types of foods as self-reported by obese and overweight people with HIV according to the National Food and Nutrition Research Institute INRAN questionnaire; Figure S2: Results of the international physical activity questionnaire (IPAQ) in people with HIV diagnosed with overweight (panel A) or obesity (panel B). Figure S3: Results of the Pittsburgh Sleep Quality Index (PSQI questionnaire) in people with HIV diagnosed with overweight (panel A) or obesity (panel B). Values ≥ 5 indicate poor sleep quality.
