# Supplementary material for: Weight Gain in Overweight and Obese People with HIV—The OBHIV Cohort
Source: J Clin Med. 2024 Feb 21;13(5):1211. doi: 10.3390/jcm13051211 (PMC10932370; doi:10.3390/jcm13051211)

Table S1: Blood parameters of glucose and lipid metabolism and lifestyle habits of the OBHIV (Overweigh and oBese people living with HIV) cohort and comparison of people diagnosed with overweight (n= 214) or obesity (N=107).

**Participants n (%)**	**Total** **n=321 (100)**	**Overweight** **n=214 (66.7)**	**Obese** **n=107 (33.3)**	**P value**
Blood parameters of glucose and lipid metabolism				
Total cholesterol (2021); mean (± SD)	191 (± 40)	194 (± 40)	185 (± 40)	0.07
LDL-c (2021); mean (± SD)	117 (± 34)	119 (± 33)	113 (± 35)	0.16
HDL-c (2021); mean (± SD)	51 (± 14)	52 (± 15)	50 (± 13)	0.22
TC/HDL (2021); mean (± SD)	3.98 (± 1.18)	3.99 (± 1.16)	3.96 (± 1.22)	0.86
Triglycerides (2021); median (IQR)	119 (85-167)	113 (85-162)	125 (83-176)	0.35
Fasting glucose* (2021); mean (± SD)	97 (± 17)	96 (± 18)	98 (± 14)	0.49
Lifestyle habits, n (%)				
**Current smoker**	125 (38.9	79 (36.9)	46 (43.0)	0.31
**Former smoker**	79 (24.6)	21 (19.6)	21 (19.6)	
**Current alcohol abuse**	124 (38.6)	84 (39.3)	40 (37.4)	0.87
**Former alcohol abuse**	10 (3.1)	6 (2.8	4 (3.7)	
**Current IDU**	7 (2.1)	6 (2.8)	1 (0.9)	0.53
**Former IDU**	53 (16.5)	36 (16.8)	17 (15.9)	

* Non-diabetic people. HDL-c: HDL cholesterol, IDU: intravenous drug user; IQR: interquartile range; LDL-c: LDL cholesterol; n: number of observations; SD: standard deviation; TC total cholesterol.

Table S2. Immuno-virological variables and antiretroviral treatment history in the OBHIV (Overweigh and oBese people living with HIV) cohort and comparison of characteristics of people diagnosed with overweight (n= 214) or obesity (N=107).

Participants n (%)	Total n=321 (100)	Overweight n=214 (66.7)	Obese n=107 (33.3)	P value
**Copy Years Viremia (log_{10} copies/mL)**	2.84 (1.23-4.18)	2.55 (1.20-4.04)	3.29 (1.26-4.52)	0.14
**Zenith HIV-RNA (log_{10} copies/mL)**	4.76 (3.60-5.32)	4.69 (3.09-5.26)	4.87 (3.98-5.48)	0.053
**Nadir CD4+ (cells/mm^3)**	220 (88-338)	236 (106-355)	197 (64-326)	0.13
**Years of HIV infection**	17 (8-26)	16.5 (8-26)	17 (8-25)	0.82
**Months of NNRTI**	37 (0-84)	36.5 (0-83.5)	38 (0-85)	0.88
**Months of PI exposure**	9 (0-91)	18 (0-82.5)	0 (0-88)	0.21
**Months of INSTI exposure**	20 (0-53)	20.5 (0-53)	19 (0-52)	0.98
**Months of TDF exposure**	71 (13-125)	63 (12-122.5)	84 (13-127)	0.65
**Months of TAF exposure**	0 (0-46)	0 (0-41.5)	0 (0-47)	0.48
**Months of ABC exposure**	0 (0-15)	0 (0-22)	0 (0-0)	0.31
**Months of XTC exposure**	119 (71-182)	120 (71-183.5)	110 (75-176)	0.89

All variables are expressed as median (interquartile range). ABC: abacavir; INSTI: integrase inhibitors; n: number of observations; NNRTI: non-nucleoside reverse transcriptase inhibitors; PI: protease inhibitors; TAF: tenofovir alafenamide; TDF: tenofovir disoproxil fumarate; XTC: lamivudine/emtricitabine.

Figure S1. Number of servings/week consumed for different types of foods as self-reported by obese and overweight people with HIV according to the National Food and Nutrition Research Institute INRAN questionnaire.

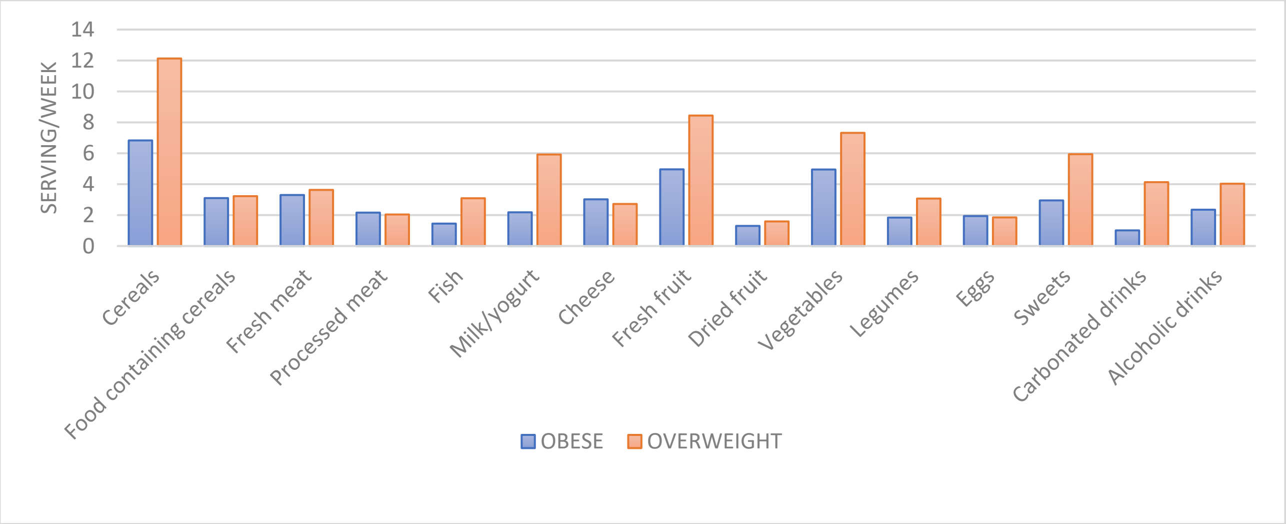

Figure S2. Results of the International Physical Activity Questionnaire (IPAQ) in people with HIV diagnosed with overweight (panel A) or obesity (panel B).

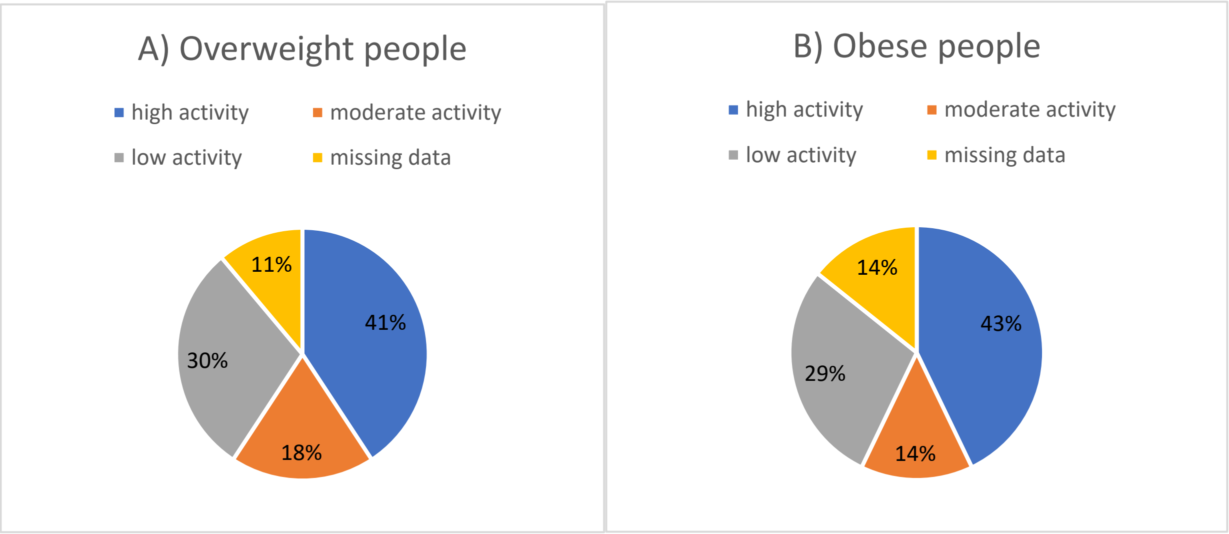

Figure S3. Results of the Pittsburgh Sleep Quality index (PSQI questionnaire) in people with HIV diagnosed with overweight (panel A) or obesity (panel B). Values ≥ 5 indicate poor sleep quality.

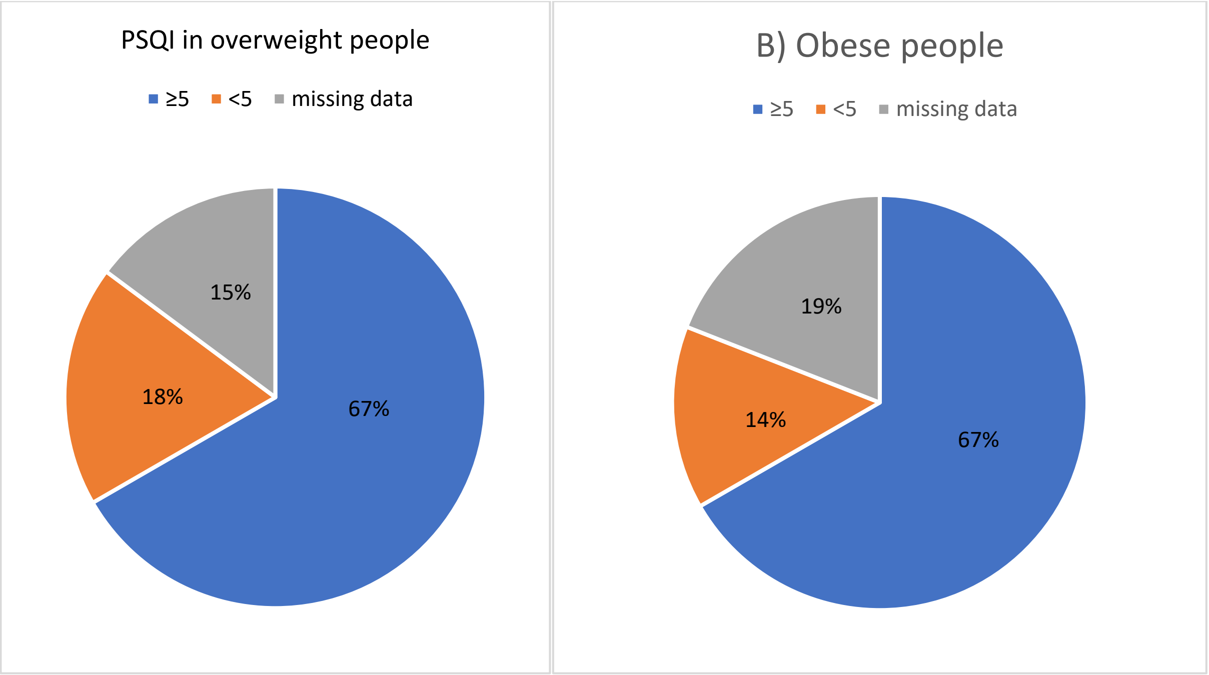

Supplement: Supplementary file 1 [file jcm-13-01211-s001.zip › jcm-2820945-supplementary.pdf]
